# Biosensors as Nano-Analytical Tools for COVID-19 Detection

**DOI:** 10.3390/s21237823

**Published:** 2021-11-24

**Authors:** Anchal Pradhan, Preeti Lahare, Priyank Sinha, Namrata Singh, Bhanushree Gupta, Kamil Kuca, Kallol K. Ghosh, Ondrej Krejcar

**Affiliations:** 1 Center for Basic Sciences, Department of Chemistry, Pt. Ravishankar Shukla University, Raipur 492010, India; anchalpradhan1010@gmail.com (A.P.); preetilahare22@gmail.com (P.L.); priyanksinha6322@gmail.com (P.S.); kallolkghosh@gmail.com (K.K.G.); 2 Ramrao Adik Institute of Technology, DY Patil University, Nerul, Navi Mumbai 400706, India; 3 Department of Chemistry, Faculty of Science, University of Hradec Kralove, Rokitanskeho 62, 50003 Hradec Kralove, Czech Republic; 4 Biomedical Research Center, University Hospital, Sokolska 581, 50005 Hradec Kralove, Czech Republic; 5 School of Studies in Chemistry, Pt. Ravishankar Shukla University, Raipur 492010, India; 6 Center for Basic and Applied Research, Faculty of Informatics and Management, University of Hradec Kralove, Rokitanskeho 62, 50003 Hradec Kralove, Czech Republic; ondrej.krejcar@uhk.cz

**Keywords:** nanobiosensor, COVID-19 detection, optical, electrochemical, smart and wearable, piezoelectric, RT-LAMP

## Abstract

Selective, sensitive and affordable techniques to detect disease and underlying health issues have been developed recently. Biosensors as nanoanalytical tools have taken a front seat in this context. Nanotechnology-enabled progress in the health sector has aided in disease and pandemic management at a very early stage efficiently. This report reflects the state-of-the-art of nanobiosensor-based virus detection technology in terms of their detection methods, targets, limits of detection, range, sensitivity, assay time, etc. The article effectively summarizes the challenges with traditional technologies and newly emerging biosensors, including the nanotechnology-based detection kit for COVID-19; optically enhanced technology; and electrochemical, smart and wearable enabled nanobiosensors. The less explored but crucial piezoelectric nanobiosensor and the reverse transcription-loop mediated isothermal amplification (RT-LAMP)-based biosensor are also discussed here. The article could be of significance to researchers and doctors dedicated to developing potent, versatile biosensors for the rapid identification of COVID-19. This kind of report is needed for selecting suitable treatments and to avert epidemics.

## 1. Introduction

The whole world is facing a deadly viral disease named COVID-19 caused by a novel corona virus—i.e., severe acute respiratory syndrome coronavirus-2 (SARS-CoV-2), first reported in December 2019 in Wuhan, China [1]. The World Health Organization (WHO) declared the outbreak of COVID-19 a global public health emergency of international concern on 20 January 2020 [2]. As per WHO data, more than 200,840,180 confirmed cases have been reported with 4,265,903 deaths worldwide as of 6 August 2021 [3]. After combating the first wave of COVID-19, many countries faced a more severe second wave of the pandemic. Due to the lack of appropriate treatment and diagnostic systems, the SARS-CoV-2 epidemic became more serious as it continued spreading over the world. Similar viruses have caused epidemics before: SARS-CoV in 2003 and Middle East respiratory syndrome (MERS-CoV) in 2012 [4]. The genome of the new COVID-19 virus has been found to be 80% similar to that of SARS-CoV, hence being named SARS CoV-2 [5]. The genetic material of SARS-CoV-2, SARS-CoV and MERS are RNA, so they are called RNA viruses. RNA viruses are more infectious than DNA viruses, as they transmit infections to cells by inserting RNA, which rapidly duplicates and transcribes viral proteins in the host cells [6]. This property of RNA viruses makes it very difficult to spot an RNA virus at an initial phase of the infection.

The current diagnostic techniques used for COVID-19 are CT scans, RT-PCR, serology tests, antigen tests, etc. The CT scan was the first technique used for the diagnosis of patients with SARS-CoV-2. The CT scans of their chests were compared with those of healthy lungs [7]. Bhanushree et al. discussed the diagnosis techniques, epidemiology and pathogenesis of the causative agents of the pandemic [8]. According to WHO guidelines, an infection by SARS-CoV-2 should be confirmed by detecting a unique RNA sequence. RT-PCR is the technique through which RNA sequences are amplified; it is used for the detection of COVID-19 [9]. This relies on complex devices and skilled operators. RT-PCR detection is slow, laborious and expensive. Researchers are enthusiastically working on the advancement of various diagnostic techniques to overcome several problems and limitations related to PCR-based techniques to develop low-cost, reliable and rapid detection methods for SARS-CoV-2.

There is a need to develop a sensing device which is less time consuming, cheap, easily accessible to all and efficient. In this regard, biosensors are ideal for providing continuous and real-time detection [10,11] via nanomodification, which can be considered a new analytical tool for the diagnosis and detection of SARS-CoV-2. Over past two decades, nanoanalytical tools and biosensors have had enormous developments in terms of low cost, ultrasensitive and early detection tools. A biosensor is an analytical device or can be defined as a bioreceptor which can measure and transduce a physical signal—an electrical, mechanical, optical or thermal one—produced from a biological change [12,13]. Nanobiosensors are antibody-based or DNA-based and allow optical, electrochemical, or field effect transistor (FET)-based transduction. The use of nanobiosensors may conquer some of the challenges and limitations of biosensing technology using novel nanomaterial. As the name suggests, the size of the nanomaterial should be within 1–100 nm. They are designed to show novel characteristics as compared to substances without nanoscale features, such as better conductivity; increased strength; and unique thermal, optical, magnetic and chemical properties. Diagnostic methods based on nanobiosensors have the advantages of reproducibility, suitability for mass production, suitability for placement of enzymes, the possibility of miniaturization, low costs, no need for calibration, reduced power consumption due to voltage reduction, reproducibility, high signal-to-noise ratios, rapidity and label-free recognition [12,13,14].

In this review, we have focused on the various types of nanobiosensors as nanoanalytical tools for COVID-19 detection. We specifically report on optical biosensors, electrochemical biosensors, smart and wearable biosensors, piezoelectric biosensors, RT-LAMP biosensors and other biosensors (pathsensor, etc.). It is expected that our review on nanobiosensors will provide exciting information for the future advancement of new nanobiosensor-based diagnostic devices for COVID-19 detection, prevention and control.

## 2. Discussion

### 2.1. Challenges with Traditional Methods

#### 2.1.1. Chest CT

It is one of the techniques used for the diagnosis of diseases by providing images of various sections of chest using X-ray radiation. Currently, chest CT scans for the diagnosis of COVID-19 have not been suggested by the international radiological guidelines [15,16]. The images of chest CT scans vary with the extent of the disease, period of scanning, age of patient, current immune response and drugs administered [17]. When a patient with COVID-19 is IgG and IgM positive, we know the immune system has speedily developed antibodies against the disease and that it started more than 14 days ago. CT scans are efficient for symptomatic patients, as they can image the lung variations. For asymptomatic individuals, CT has a significant level of inaccuracy [18]. The use of CT for diagnostic COVID-19 imaging can be inaccurate due to other conditions, such as pneumonia. Indeed, for COVID-19, CT has a very low specificity (only 25%). Furthermore, frequent irradiation can be damaging to long-term health [19]. A CT system is costly equipment that requires an expert in order to operate it and evaluate the data. The other disadvantages of chest CT scanning are the high cost each use, the possibility of misinterpretation, ineffectiveness for asymptomatic conditions and excessive radiation exposure [20,21].

#### 2.1.2. RT-PCR

Managing coronavirus infections and identifying probable viral sources is crucial; thus, quick diagnosis of sick patients is necessary. Serology, virus population and antigen identification are all used in many early diagnosis techniques [22]. In emergency diagnostic virology, real-time RT-PCR is routinely employed, which is a molecular technique. If a coronavirus has been identified as the causative agent of respiratory distress by a molecular diagnostic test, the species must then be identified. If SARS-CoV-2 is present in the lower respiratory tract or in samples from a throat swab, real-time RT-PCR fluorescence can be used to identify it. RT–PCR entails converting SARS-CoV2 viral RNA to cDNA using a reverse transcriptase enzyme, followed by amplification of reverse transcribed cDNA. These primers are used to perform SARS coronavirus-specific amplification. In the early phases of a pandemic, RT–PCR has several advantages and distinctive qualities, making it a star in the diagnostic field. RT-PCR, for example, can be used to identify a viral genome early in the course of infection. RT-PCR is also exceedingly specific, sensitive and quantitative because it can assess the patient’s viral load [23]. The use of costly reagents and fairly sophisticated methods are some downsides of this technology. 

To obtain great sensitivity, high precision between primers and models is essential. The approach, however, has a number of technical drawbacks, including varying temperature requirements for different reaction cycles and the need to scan a large number of samples quickly. Moreover, diagnosis of COVID-19 through RT-PCR may also result in false negative cases. Sometimes, RNA gets degraded and disintegrated in the bloodstream due to poor stability during the viral replication cycle, which makes RT-PCR-based detection difficult. Additionally, the examination of any type of body fluid cannot give us the exact information of the viral load and stage of lung infection [24]. RT-PCR is difficult to use in underdeveloped rural areas, as it requires skilled operators, sophisticated laboratories and many reagents [25]. False negative cases in PCR-based SARS-CoV-2 detection can be caused by a variety of factors. For starters, during the viral replication cycle, RNA can be degraded due to decreased stability, resulting in viral genome fragmentation in the circulation, which makes RT-PCR-based detection tough [21,26].

#### 2.1.3. Meta-Transcriptomics Next Generation Sequencing (mNSG)

The mNSG techniques are used for the viral detection. The transcriptomes can give ideal information on the gene expression and post transcriptional variations of SARS-CoV-2. For categorizing SARS-CoV-2, a small sample size of primer metagenomic sequences have been used [27,28]. It is a convenient method for detection, as it can classify rarely expressed genes, and detect the process of biosynthesis using the whole meta-transcriptome (thereby including non-coded RNAs). However, this technique also has several disadvantages—for instance, unstable mRNA can decompose the sample before sequencing, and also, the RNA of microbes and the RNA of host cells can be laborious to differentiate [21,29].

#### 2.1.4. Serological Tests

There are many serology-based assays designed for the detection of antibodies against SARS-CoV-2 or proteins of the virus which are available in plasma or serum. There are various commercial assays for SARS-CoV-2 detection, such as an enzyme-linked immunoassay (ELISA), a chemiluminescence immunoassay (CLIA) and a lateral flow immunoassay (LFIA). These immunoassays use immunoglobulins—i.e., IgG and IgM—which are produced in response to viral infections. IgM can be observed 10 to 30 days after initial infection, whereas IgG can be found 20 days after, in SARS-CoV-2 patients. IgM is produced rapidly but later disappears. In contrast to that, IgG can be detected for a longer period of time and also provides a superior response. There are many ELISA kits available for the detection of the spike protein and nucleocapsid protein [30]. These methods are high in sensitivity; however, they have many drawbacks, such as high cost, slowness, the inability to handle multiple samples and the need for trained employees [21,31].

As we can assume from the above information (Table 1), the accuracy of immunoassays for COVID-19 will change as the viral load differs during the infection. They may give false positive results due to cross-reactivity with other coronaviridae viruses which are somewhat analogous to SARS-CoV-2 [32]. Although this method is cost-effective and less time consuming than ELISA, the sensitivity is very low compared to other diagnostic methods [33]. Additionally, the antibody tests for COVID-19 show very low sensitivity as compared to the PCR test [34]. Due to the presence of a homologous protein present in the sample, a lower titer value of the antibody or low instrumental sensitivity, this method may give false results [26,35].

**Figure 1:**
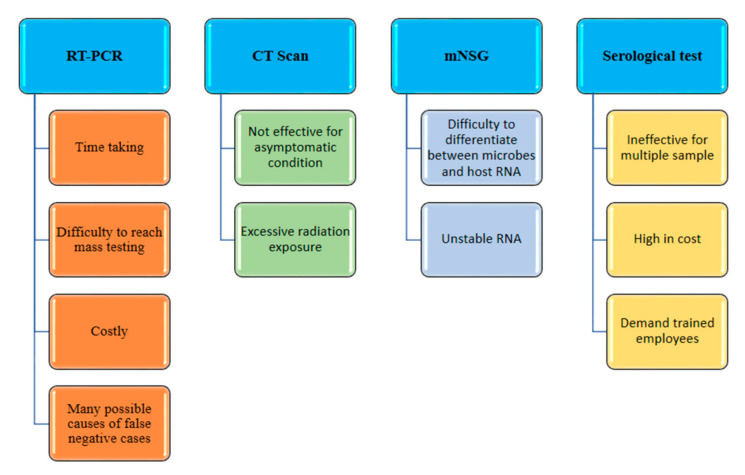
Disadvantages of traditional methods.

Despite the fact that these techniques are considered gold standards for clinical diagnosis due to their high sensitivity and specificity, they are part of centralized testing [36], which involves collecting samples from patients in a hospital and then sending them to another laboratory for analysis [37]. Furthermore, because these treatments involve hard, time-consuming and expensive procedures, they have a number of limitations in practice. The test result is usually not provided to the patient until the next day with such methods (Figure 1). As a result, decentralized testing as a point-of-care (PoC) concept is critical for the timely and accurate detection of such diseases [38,39,40] and any conditions that require further downstream studies [41,42,43,44,45].

### 2.2. Biosensors for Point-of-Care Diagnosis

**Figure 2:**
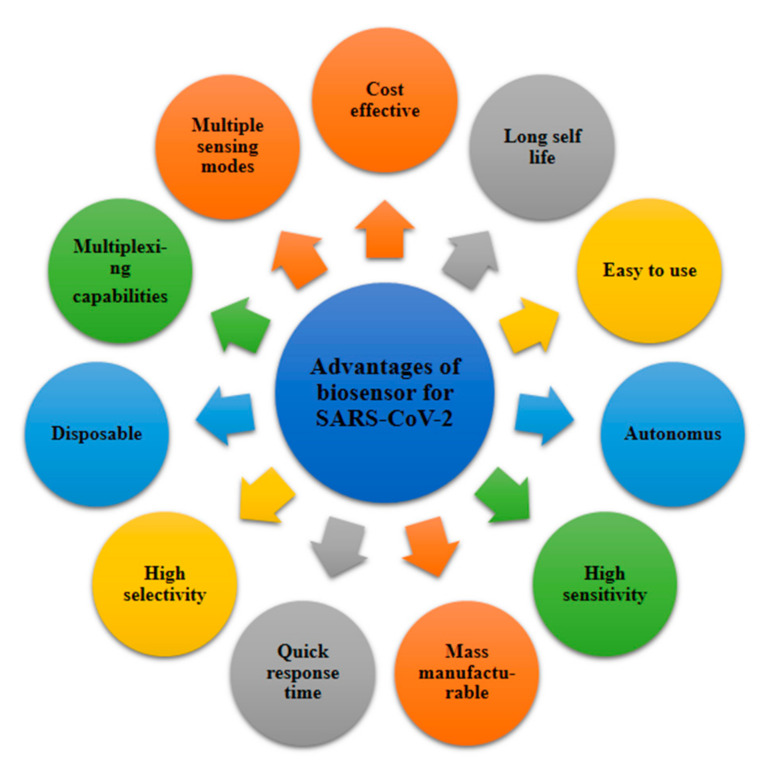
The advantages of biosensors for SARS-CoV-2.

The procedures of point-of-care are helpful for curing patients without laboratory methods. There are several advantages of point-of-care diagnosis, such as low cost, rapidity and reliable detection, which fulfils the current needs for COVID-19, and this may be the best way to solve the current situation (Figure 2). Biosensors can be used for the diagnoses. Nanobiotechnology contributes to biosensors for analytical purposes. By using a specific transducer in a biosensor, it can serve as a great technique compared the other complex methods, such as CT scanning, RT-PCR, ELISA and LFA. Biosensors are analytical tools which may consist of biological substances such as nucleic acids; enzymes; cell receptors; tissues; proteins; and extracted samples, such as designed proteins, recombinant antibodies and aptamers. These materials are present alongside physical/chemical-based tranducers such as optical, electrical, piezoelectrical or electrochemical ones within the miniature system of the biosensor [46]. An immunosensor based on the electrochemical method is a biological electroanalytical device which is created from gold nanoparticles, and therefore, it provides extraordinary properties, such as low cost, high sensitivity and miniaturization [47]. Based on that method, nanobiosensors have been designed for the detection of COVID-19, which may remove the limitations and challenges which were found in earlier detection techniques, such as CT scanning, RT-PCR and ELISA. The size of such nanomaterials is 1–100 nm. These materials have great attributes, such as optical, magnetic, chemical and thermal responsiveness and high strength. Such materials for biosensors can be metal nanoparticles, magnetic nanoparticles, carbon nanostructures or quantum dots [48,49,50,51,52,53,54]. In this regard, several nanobiosensors have been used for COVID-19 detection (Table 2) [21,55].

### 2.3. Principles of Biosensors

**Figure 3:**
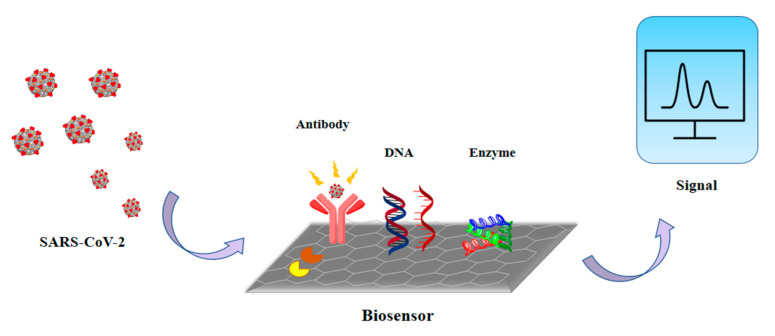
The working principle of biosensors.

**Figure 4:**
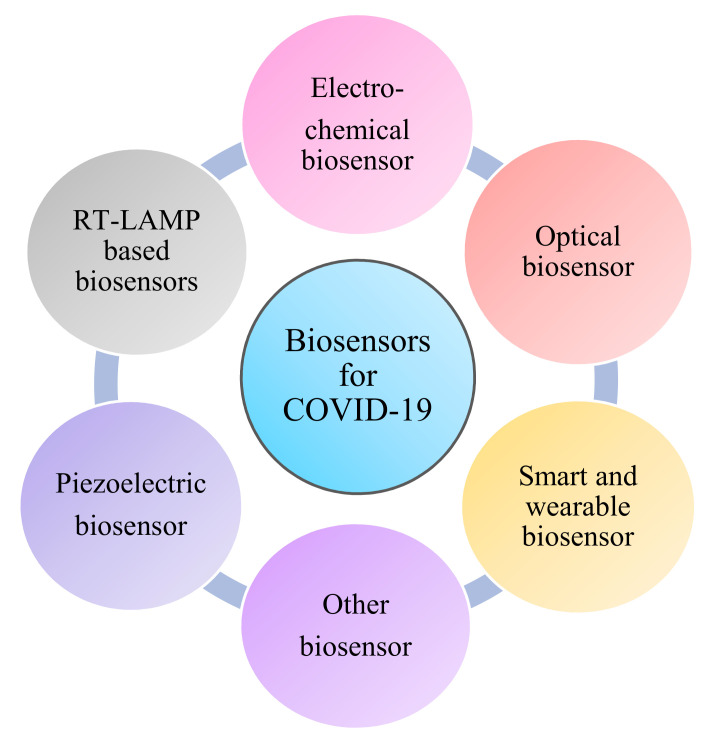
Types of biosensor for the detection of COVID-19.

For the public and non-professionals, biosensors can provide sensitive, simple equipment which can be used to detect the presence of analytes. A biosensor reacts with the analyte that is present in the sample by using its combination of transducer and a biometric system which shows the concentration of the analyte via an electronic signal (Figure 3). The detection of specific viral molecules, such as a surface antigen or protein [56,57,58], and nucleic acid (NA) sequence detection, are the two main tactics for biorecognition [59,60,61]. When a nanotechnology-based biosensor is labeled with antibodies, NA probes or other specific molecules with affinity for the target structures can attach, and the sensitivity and specificity become higher [62]. The classifications of biosensors are based on the signal sensing methods and principles of transduction used (Figure 4).

### 2.4. Techniques and Types of Biosensor

#### 2.4.1. Optical Biosensors

**Figure 5:**
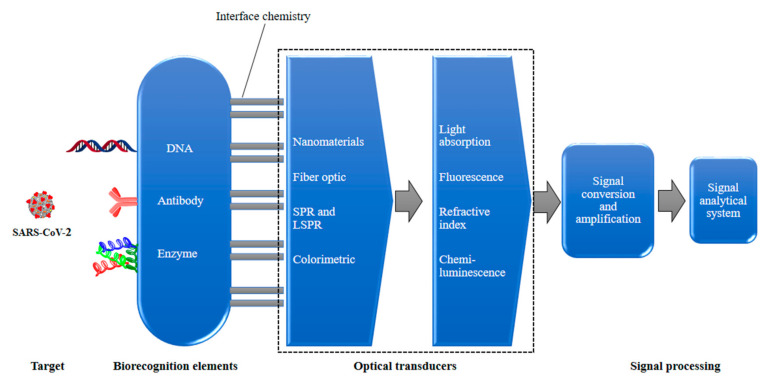
A schematic of an optical biosensor.

Optical biosensors are used as diagnostic tools [63] for respiratory virus infections [64]. This is possible due to the extraordinary attributes of optical biosensors, such as high sensitivity, being label-free, robustness, immunity to electromagnetic interference, having computable optical outputs, being amenable to miniaturization, integration capabilities, portability [65,66], multiplexing capability and providing concurrent detection of various targets (Figure 5). Therefore, optical biosensors are suitable for the point-of-care zone [67,68]. Researchers from Empa and ETH Zurich (Zürich, Switzerland) were successful at developing an optical sensor for SARS-CoV-2 [69]. Particularly, highly effective optical biosensor-based detection of SARS-CoV-2 [70] has been demonstrated with surface plasmon resonance [71,72,73,74] and fluorescence [75]. Notably, it was found that fluorescence can detect the 3a protein (3a gene encodes a non-structural viral protein) of SARS-CoV-2 (Waye et al.) [76]. In particular, when an optical biosensor is integrated with the surface plasmon resonance method, the resulting technique is useful for prompt diagnosis of SARS infection, more so than enzyme-linked immunosorbent assays (ELISA). A fiber-optic-enabled biosensor based on localized surface plasmon coupled fluorescence (LSPCF) can sense the recombinant N protein (SARS-CoV-N) by using AuNPs [77]. It was observed that a viral stock as small as 106 particles/mL can be detected by using a fiber-optics-based nano-enabled biosensor within 15 min [78,79]. These surveys demonstrate that viral respiratory infections can be diagnosed rapidly and promptly by using nanomaterial-enabled biosensors [64].

**Figure 6:**
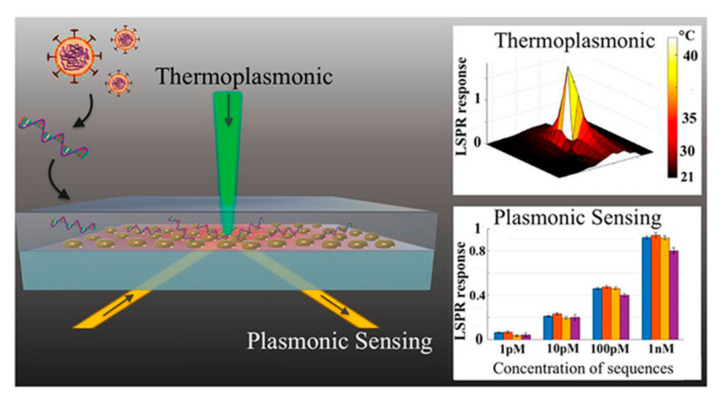
LSPR detection of nucleic acid sequences from SARS-CoV-2. The schematic shows the architecture of the LSPR substrate consisting of gold nanoparticles. Light is illuminated on the substrate for generation of local heat and detection of binding nucleic acid binding events. The graph also shows the LSPR response to the theroplasmonic effect and toward the detection of nucleic acid sequences at low concentrations. Reproduced with permission from [88] (further permission related to the material excerpted should be directed to the ACS). Direct link: https://pubs.acs.org/doi/10.1021/acsnano.0c04421 (accessed on 9 November 2021).

Furthermore, localized surface plasmon resonance (LSPR) biosensing systems consist of optical biosensors which are suitable for various classes of analytes of clinical interest [80]. Molecular binding and refractive index change LSPR sensing systems exhibit high sensitivity to local variations, owing to the enriched plasmonic field in the locality of a nanostructure [81]. Thus, for label-free and real-time detection of micro and nanoscale analytes. LSPR is an ideal candidate [82,83]. A promising and alternate solution for COVID-19 diagnosis is provided by a dual-functional plasmonic biosensor linking LSPR sensing transduction and the plasmonic photothermal (PPT) effect (Figure 6). Through nucleic acid hybridization, one can perform sensitive detection of select sequences from SARS-CoV-2 using two-dimensional gold nanoislands (AuNIs) functionalized with complementary DNA receptors. With the two-dimensional distribution of nanoabsorbers, (AuNIs), the plasmonic chip is capable of transducing via in-situ hybridization and generating the local PPT heat for highly accurate and sensitive detection of SARS-CoV-2 [84]. A quick immunoassay for instantaneous recognition of antibodies against COVID-19 nucleocapsid protein (Nuc) and three COVID-19 spike protein antigens (receptor binding domain, RBD; spike S1 fragment; spike S1S2 extracellular domain) is provided by a multiplexed grating-coupled fluorescent plasmonics (GC-FP) platform. Using serum, it reached 100% sensitivity and specificity for detecting prior COVID-19 infection, and for dried blood spot samples (DBS) it showed sensitivity as high as 86.9% and 100% specificity [85]. Moreover, an optomagnetic biosensor which is based on homogeneous circle-to-circle amplification (HC2CA) [86] was applied for the detection of synthetic complementary DNA (cDNA) and RNA-dependent RNA polymerase (RdRp) coding sequences of SARS-CoV-2, having a limit of detection at the femtomolar level [87]. Hence, this can be fabricated for the detection of SARS-CoV-2 in the air and in real time.

#### 2.4.2. Electrochemical Biosensor

**Figure 7:**
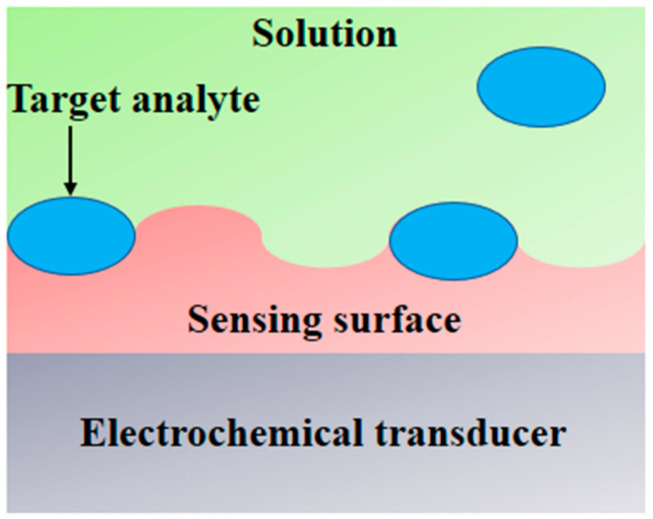
A schematic of an electrochemical biosensor.

In interdisciplinary applications, we have seen a significant rise in electroanalytical methods, and exceptional improvements in the performances and designs of electrochemical devices because of the upsurge of nanotechnology. An electrochemical sensor has the ability to measure changes in potential, conductivity, current and impedance due to the recognition process happening on the sensing surface while the electrode material acts as the transducer (Figure 7) [89,90]. In the detection of different viruses and their correlated antigens and antibodies, electrochemical techniques have rapid analysis, high sensitivity and high selectivity [91]. An FET-centered electrochemical sensor is made up of a field effect transistor as a sensing surface and a transducer component, which includes a dielectric layer operationalized with particular receptors that have selective affinity for the target analyte [92,93]. On the sensing surface, when analytes are caught due to a change in FET electrical properties in the form of channel conductance or a drain-to source, the electrostatic effect is then converted into a measurable electronic signal [93]. For the electrochemical detection of coronavirus-related proteins, detection with FETs has been used, as the nature of the signal output of each field effect transistor is known [91]. To detect the SARS-CoV-2 genome, electrochemical DNA sensors have been provided, though there is a difference between label-based and label-free approaches. With the use of enzymes, metal complexes, electroactive substrates and heterocyclic dyes as the electroactive labels, label-based assays provided high sensitivity, whereas label-free detection was performed by exploiting the intrinsic electroactivity of the DNA nucleobases [91]. An electrochemical sensor chip was developed which has many good attributes—i.e., it works at a good speed (less than 5 min), is easy to operate, is low cost, is quantitative and is paper based. It was developed for enabling digital detection of SARS-CoV-2 genetic material. Some significant obstacles, such as low hybridization process efficiency and poor sensitivity on the surfaces of bulk electrodes, have been encountered by nucleic acid-based biosensors designed for analyzing clinical samples. Such problems are solved through the use of a gold nanoneedle structured electrode, as it increases the surface area of the electrode, which enhances the working capability of the biosensor [94]. To target the viral nucleocapsid phosphoprotein (N-gene), the biosensor uses gold nanoparticles (AuNPs), capped with highly selective antisense oligonucleotides (ssDNA). The sensor showed 100% sensitivity, accuracy and specificity [95]. For the selective detection of SARS-CoV-2, a In_2_O_3_ nanowire field effect transistor (FET) modified (revise) with the antibody mimic protein fibronectin (Fn) can be used [96]. A low-density carbon nanotube FET (CNTFET) was fabricated to enhance its detection performance for the N protein of SARS-CoV-2 [97].

**Figure 8:**
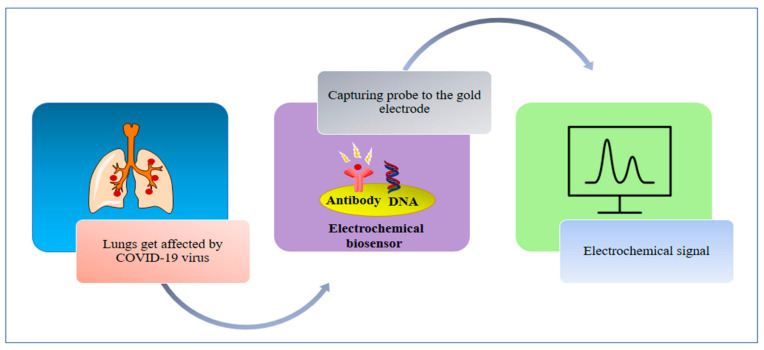
A schematic of the mechanism of electrochemical biosensors.

Additionally, for the selective detection of reactive oxygen species (ROS) in sputum samples, a simple electrochemical sensor was designed. It is made up of a disposable sensor and an electrochemical board which gives an output automatically. The sensor consists of multi-walled carbon nanotubes (MWCNTs) which are on the top of the steel needles in a triangular structure of three electrodes which are 3 mm from each other—namely, the reference, counter and working electrodes. It detects the ROS level in the sputum of a COVID-19 infected patient as an indicator or lung dysfunction which is induced by the virus’s forcing of mitochondrial ROS overproduction (Figure 8) [98].

For the identification of the S1 functional subunit of the spike protein of SARS-CoV-2, a biosensor based on the bioelectric recognition assay (BERA) was developed. When the virus gets in contact with the host, this S1 subunit interacts with the angiotensin-converting enzyme-2 (ACE-2) receptor in the host [99]. The interaction between the S1 functional subunit and antibody results in bioelectric property changes. With a detection limit of 1 fg/mL, this technique provides a rapid response against SARS-CoV-2 nucleocapsid protein, and no cross-reactivity was perceived [100]. A capable detection tool has emerged which is called the microfluidic paper-based analytical device (µPAD), and to improve its utility and performance, electrochemical impedance spectroscopy (EIS)-based sensing was used, as it shows label-free operability and high sensitivity. Improving the EIS biosensing of µPADs has not been well explored. To enhance the performance of paper-based EIS nanobiosensors, a working electrode was used along with vertically grown zinc oxide nanowires (ZnO NWs). In human serum samples this nanobiosensor can differentiate the concentrations 1 μg mL^−1^, blank, 100 ng mL^−1^ and 10 ng mL^−1^ of IgG antibody (CR3022) against SARS-CoV-2 [101]. By using calixarene-functionalized graphene oxide for targeting the RNA of SARS-CoV-2, ultrasensitive electrochemical detection technology was made. Without any reverse transcription and amplification of nucleic acids, this can be performed using a portable electrochemical smartphone. During actual testing and in silico analysis, this biosensor showed high selectivity and specificity. Thus, for SARS-CoV-2 detection, this convenient, ultrasensitive and accurate assay provides a potential method for point-of-care testing [102]. Likewise, clustered regularly interspaced short palindromic repeats (CRISPR)-associated (Cas) enzyme technology can detect specific gene sequences of COVID-19 within one hour, with detection limits between 10 and 100 copies per microliter; the technology is targeted amplification based [103]. Recently, a group of researchers reported their intent to fabricate an electrochemical CRISP biosensor which is amplification-free that works via nanoalteration of the surface of the electrode to enhance the signal [55].

**Figure 9:**
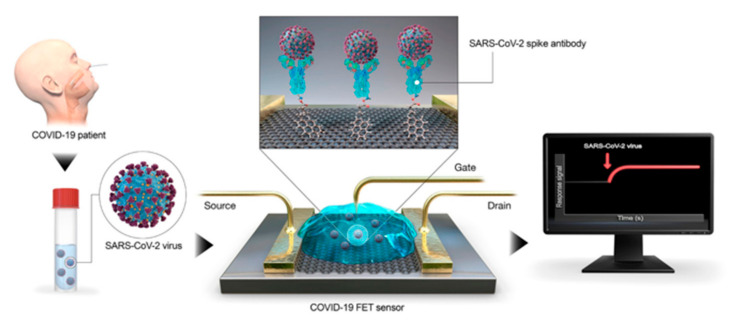
Detection of SARS-CoV-2 using FETs: The schematic shows a collection of biological samples from a patient and their application to the graphene-based sensing area of a FET biosensor. Binding events associated with the SAR-CoV2 virus can be captured by the sensor in real time. Reproduced with permission from [88] (further permission related to the material excerpted should be directed to the ACS). Direct link: https://pubs.acs.org/doi/10.1021/acsnano.0c04421 (accessed on 9 November 2021).

Moreover, graphene-based biosensors are useful for testing and advanced detection of blood glucose, respiration rate, real-time body temperature, blood pressure, virus, small molecules, protein interactions and allergen sensing [104,105]. For biosensors, graphene-based nanomaterials are the most attractive materials to come out in the last few decades due to their cost-effectiveness, high affinity and ease of fabrication [106,107,108]. Recently, a transistor-based biosensor has been successfully developed that detects SARS-CoV-2 (spike protein). The biosensor was fabricated using coated graphene sheets of field-effect transistor (FET) with a specific antibody (Figure 9) [109]. For capturing viruses, graphene and its derivative show good integrity [110,111]. FET-based biosensing devices have advantages over other diagnostic methods which are available currently. They have the potential to make highly sensitive and instantaneous measurements by using small amounts of analytes [112,113]. FET-based biosensors are known for having potential and utility in clinical diagnosis, on-sight detection and point-of-care testing. To detect SARS-CoV-2 RNA in human throat swab specimens, an unamplified and rapid nanosensing platform was developed. A graphene field-effect transistor (G-FET) sensor was developed which was gold nanoparticle (AuNP) decorated. On the surfaces of AuNPs, complementary phosphorodiamidate morpholino oligo (PMO) probes were immobilized. This sensor leads to a low background signal, as the PMO is highly sensitive to SARS-CoV-2 RdRp [114]. When a graphene field effect transistor is coupled with a CRISPR-Cas9-based biosensor, it will be able to detect unamplified target genes, and thus, it could be considered for viral target, such as of the nucleic acids of SARS-CoV-2 [115,116].

#### 2.4.3. Smart and Wearable Biosensors

Akyildiz et al. put forward the concept of Internet of Bio-Nano Things (IoBNT) for POC diagnostics for nanoscale sensing devices which provide health information to an external health provider through the Internet [117,118]. Moreover, nanomaterial-based electrodes, when connected to electronic devices, can be used for health monitoring purposes by reading the wireless communication [119] of the output signal and processing this signal in a smart phone or computer [118]. Therefore, smartphone-based healthcare operates well for data analysis, data recording and data sharing [120,121,122]. Particularly, artificial intelligence (AI) could be used as a tool for preventing the spreading of SARS-CoV-2 [123]. Thereby, AI-assisted IoT medical-based information can be used for diagnosis and monitoring of COVID-19 [124] in a personalized manner by involving a smartphone [125]. This method is already adopted by US health institutes to monitor patients at home for avoiding the spread of COVID-19 [126,127,128]. The demand for smart sensing of AI-assisted IoT with nanoenabled SARS-CoV-2 biosensors at the personal level has been raised due to the wireless systems for tracing the population and because a SARS-CoV-2 infection can be asymptomatic [123,129]. Likewise, electrochemical nanoenabled sensors when fabricated in mobile health platforms provide ultra-sensitive and rapid testing by transferring the data to user through Bluetooth. [124]. Even more, the smart management of SARS-CoV-2 can be achieved in personalized manner by selecting specific anti-SARS-CoV-2 protein antibodies through miniaturized interdigitated electrode (IDE)-based SARS-CoV-2 biosensors, which can give selective and sensitive detection within 30–40 min; and by understanding the therapies, disease progression and relationship between SARS-CoV-2 level and pathogenesis. Furthermore, an algorithm which supports AI will be useful for predicting the needs for safe social distancing practices [124], lockdowns and targeted testing; and for selection of the best therapy among the available vaccines and drugs [130]. Artificial intelligence (AI) is also used to detect the effect of COVID-19 in exhaled breath through a hand-based breathalyzer system [131]. In this system, AuNPs with organic ligands generate electric resistance due to compression and expansion of a nanomaterial film which is based on the chemical reaction of gases present in exhaled breath, water vapor and volatile organic compounds. This diagnostic procedure was noted to be highly specific, rapid and simple when testing for COVID-19, compared to other respiratory infections [132]. Additionally, several applications have been constructed for mass screening of COVID-19—for example, aarogya setu for observing the current and potential hotspots [133]—and social-media platforms have been used to spread instant information [134]. Hence, we can perform bioinformatics by considering several machine learning, data processing and mobile healthcare platforms used for COVID-19 and also consider mental health during the pandemic [135,136,137]. Hence, this technology provides smart healthcare by performing several programming operations for the management of the COVID-19 pandemic [130].

**Figure 10:**
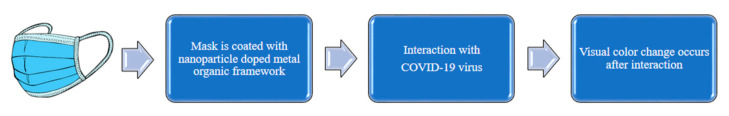
Workflow of a wearable nanobiosensor.

In traditional laboratory-based diagnostic tests, laborious sample processing procedures were involved. To encourage non-invasive measurements, continuous monitoring and more efficiency, recently, wearable sensors have been getting more consideration [130,138,139]. As compared to blood, biological samples such as tears and sweat may have more selective detection. For addressing mass-level screening, wearable sensors are efficient and offer point-of-care diagnosis, which is important in the prevention of wide expansion of a disease [100]. Before the appearance of clinical symptoms of COVID-19, an android and some wearables could predict the alterations in physiological status [140]. Researchers have created a software-based monitoring system which can be used to detect COVID-19 by considering respiratory and heart functions, as these are highly related to infection with this virus [141,142]. This software-based sensor uses skin fit wearable devices, such as electrocardiogram (ECG) sensors for diagnosis of heart function, and pulse oximeters for the diagnosis of shortness of breath and oxygen saturation levels of the patients [143]. Additionally, an increase in the usage of smart watches, including WHOOP, Fit bit, VivaLNK and Amaze fit etcetera, is noticeable these days. For the purposes of measuring temperature, heart rate and blood pressure, these wearables (optical sensors and accelerometers) can be used [144]. For complete measurements of body temperature, respiration related features and heart activity-related vital parameters (heart rate, heart sound and cardiac amplitude), a chest mounted patch sensor was developed by Rogers and his group, in collaboration with the US Department of Health and Human Service’s Biomedical Advanced Research and Development (BARDA) and Sonica Health. The patch sensor is put in direct contact with the skin at the base of the neck, and it includes a temperature sensor and an accelerometer. With 50 subjects, the initial phase of testing the patch sensor was carried out, and the aftermath was that the changes in the respiratory parameters were related to each other, so it made it easy to know the prognosis of COVID-19 infection [145]. Further, the patch sensor provides more coziness to the patient, and this encourages applications of it in mass-level testing. For early detection, a face mask for sensing applications could be vital. To deal with SARS-CoV-2, wearable masks were made with a metal–organic framework that displays changes in color. Chemisorption or physisorption was utilized when nanoparticles were doped in a nanoporous matrix, such as a metal–organic framework (MOF). Then the doping of nanoparticles was performed. Due to changes in optical properties when nanoparticles interact with the virus, as the outcome, there is a visible color change (Figure 10) [146]. Moreover, a functionalized immunosensing chip with specific monoclonal antibodies in opposition to SARS-CoV-2 spike protein [147] can be used, but the current immunosensors are not suitable due to the small size of SARS-CoV-2 (around 100 nm): the viral particles cannot contact the surface of sensor in the low-Reynolds-number hydrodynamic conditions [148]. Smart nanostructures can be used to detect the virus in environmental air samples due to their large surface areas [149,150,151,152,153,154,155]. It has been proven that SARS-CoV-2 can spread through breath [156]. Hence, intelligent face masks are being developed, where a high density of conductive nanowire arrays equal in size to the virus, a miniaturized impedance circuit and a nanoimpedance immunosensor are implanted. Nanowire arrays are attached to the face mask via a flexible plastic polymer constructed with a nanoscale soft printing mechanism, which decreases its cost. When viral targets are detected through biological components, impedance signals will be changed, and a wireless signal will be transmitted to a smartphone through Bluetooth and a miniaturized impedance circuit. The face mask as a POC tool is used as a source of viral samples. In this case, it has highly concentrated nanowires to effectively catch and accumulate the exhaled viral aerosols. In simulated breath aerosols and with a diluted aqueous solution, the POC device was used to identify spike proteins and intestinal flu virus (coronavirus mimics). With the use of nanowires, there are advantages such as ultra-low power consumption; small size; rapid response; high sensitivity; and being non-hazardous, easy to use, non-invasive, stored well and not that costly. For the management and detection of respiratory infections, a sensitive and affordable POC tool was provided as a combination of nanoscale sensors and a face mask [157].

#### 2.4.4. Piezoelectric Biosensors

**Figure 11:**
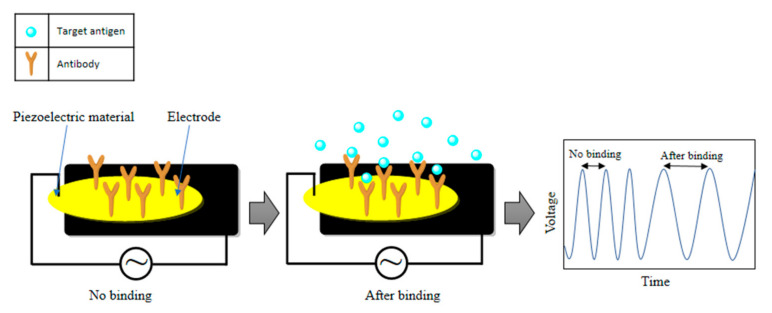
The operation principle of a piezoelectric biosensor.

Piezoelectric quartz crystal microbalance (QCM) nanobiosensors have achieved recognition for the medical applications due to their simplicity, label-free testing, flat surfaces and real-time responses [158,159,160]. According to Albano et al., detection of protein biomarkers at the pg/mL level is possible by analyzing the effect of paramagnetic nanoparticles using a piezoelectric quartz crystal nanobiosensor [161]. Particularly, a piezoelectric immunosensor can be identified as stable, effective and fast for SARS-CoV detection (Figure 11) [162]. Furthermore, QCM-based nanobiosensors can be helpful for the detection of SARS-CoV-2 from oral swabs [163]. In this method, a SARS-CoV-2 spike protein response can be identified [163] when the spike glycoprotein get linked with the platform of the sensor through an adsorption mechanism, and hence it shows high sensitivity at the ng level. Again, these biosensors are useful for SARS-CoV-2 detection [164].

#### 2.4.5. RT-LAMP Based Biosensor

**Figure 12:**
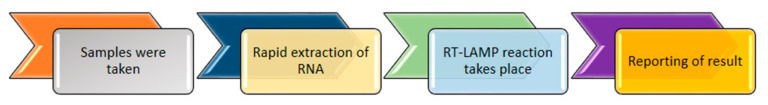
Working mechanism of the RT-LAMP assay.

The loop-mediated isothermal amplification (LAMP) technique has been used for the detection of COVID-19 (Figure 12). This technique amplifies the desired gene sequences in isothermal conditions, whereas PCR amplifies the genes at various temperatures, which limits its applicability for resource-limited laboratories. Additionally, a reverse transcription-loop mediated isothermal amplification-nanoparticle-based biosensor (RT-LAMP-NBS) was developed by using colorimetric sensing nanoparticles for visual detection, and it offers effective and easy use for clinical laboratories. This apparatus requires a heating mantle to maintain a steady temperature of 63 °C for around 40 min, and its working features include two LAMP priming sets, nucleoprotein (NP) and F1ab (open reading frame (ORF) 40 1a/b) of SARS-CoV-2. The one-step RT-LAMP reaction amplifies and identifies two target genes (np and F1ab) at the same time, which adds more precision in the result. Thus, the complete procedure for diagnosis, from the sample collection to the result evaluation, takes around 1 h. It was experimentally found that oropharynx swab samples are effective for providing precise results, as they gives 12 copies per reaction in the case of SARS-CoV-2 templates, whereas non- SARS-CoV-2 templates do not allow cross-reactivity. Hence, this method can be used as a diagnostic tool for COVID-19 because of its sensitivity, simplicity, high precision and cost-effectiveness [21].

#### 2.4.6. Other Biosensors

Path-sensors have applications for detecting pathogens in the air. Additionally, Path-sensors Inc. developed a fast, highly sensitive and effective biosensor in March 2020 which is called CANARY^TM^ [21] for the aerosol detection of SARS-CoV-2. This biosensor is based on a genetically modified immune cell able to detect and bind to a specific target. It amplifies the signal coming from the cell within 3–5 min. After this, the presence of a targeted pathogen can be identified by measuring the intensity of the signal which comes from the cell [55,165].

Newly, Abbott ID Now^TM^ manufactured a detection kit which is based on the loop mediated isothermal amplification (LAMP) technique. It can detect COVID-19 within 5 min by taking samples from oral swabs, nasopharyngeal ones, nasal ones, etc. In this method, fluorescent molecular beacon probes are used to identify the amplicons, and the primers are used to identify the RNA-dependent RNA polymerase (RdRp) gene sequences. These kits are present in limited amounts and consist of 24 tests, which also include swabs for collecting samples and pipettes. Food and Drug Administration—Emergency Use Authorization (FDA EUA) gave approval to this kit as a commercial product [166].

RNA sequencing has been used for the detection of COVID-19 by identifying the transposons which can disintegrate the hetero-DNA–RNA hybrids. Recently, a tool has been constructed by the researchers at the Peking University (China) for the diagnosis of COVID-19 which offers early, fast and precise data to characterize the RNA by collecting information for sequencing hetero-DNA–RNA hybrids. Tn5 transposase was used, which binds and randomly cuts [167] dsDNA along with the prime fragments of hetero-DNA–RNA hybrids which are produced by the process of reverse transcription. These prime fragments were amplified by using the polymerase chain reaction (PCR).

Recently, eCovSens was developed by Mahari et al., which is a domestic biosensor system that uses gold nanoparticles (AuNPs) and electrodes of a COVID-19 antibody and fluorine doped tin oxide (FTO), which are extremely specific to the SARS-CoV-2 spike antigen. These antigen-based sensors can detect SARS-CoV-2 antigen from 1 fM to 1 μM concentrations in ideal conditions and at 10 fM in standardized buffer within 10–30 s [21,168].

**Table 2:** Methods and properties of biosensors being used for the detection of COVID-19.

Types of Biosensors	Scheme	Nanomaterials	Detection Methods	Target	Limit of Detection	Detection Range	Sensitivity	Assay Time	Ref.
	Upper respiratory tract (URT) specimen	Gold nano-islands (AuNIs)	Plasmonic photo-thermal (PPT) and localized surface plasmon resonance (LSPR)	SARS-CoV-2 Nucleic acid	0.22 pM	0.1. pM to 1 μM	Not mentioned	No mentioned	[84]
Optical biosensor	Oro-pharyngeal swab	Gold nano-particles	Plasmon based colorimetric biosensing	N-gene of SARS-CoV-2	0.18 ng/µL of RNA	0.2–3 ng/µL.	Not mentioned	10 min	[64]
	Naso-pharyngeal sample	Gold NPs	Plasmonic effect based colorimetric biosensing	RdRp gene of SARS-CoV-2	0.5 ng	Not mentioned	Not mentioned	Approx. ~30 min	[64]
	Serum	Gold nano-particles	multiplexed grating-coupled fluorescent plasmonics (GC-FP) biosensor platform	IgG, IgM, IgA	Less than 2 ng/spot	Not mentioned	86.9%	Less than 30 min	[85]
	Blood samples	gold nano particle (AuNP)	colorimetric assay	IgG-IgM combined antibody SARS-CoV-2	Not mentioned	Not mentioned	Not mentioned	15 min	[64]
	Serum	gold nano-particle (AuNPs)	LSPCF fiber-optic enabled biosensor	recombinant N protein of SARS-CoV-N	1 pg/mL	0.1 pg/mL to1 ng/mL	Not mentioned	Not mentioned	[64]
	Serum	Lanthanide-doped poly-sterene NPs	Lateral flow immuno-assay (LFIA) based on fluorescence biosensing	Anti-SARS-CoV-2 IgG in positive sample,	Not mentioned	Not mentioned	Not mentioned	10 min	[64]
	Upper and lower respiratory specimens	Iron oxide NPs	Opto-magnetic sensing	RdRp coding sequences SARS-CoV-2	0.4 fM dynamic	10 to 105 copies	10 copies sensitive	100 min	[164]
	Clinical samples	Gold nano-particles	Antisense Oligo-nucleotides Directed Electro-chemical Biosensor Chip	nucleo-capsid phospho-protein (N-gene)	6.9 copies/μL	Not mentioned	100%	Less than 5 min	[95]
	Not mentioned	In_2_O_3_ nanowire	Metal-Oxide-Semi-conductor FET (MOSFET)	SARS-CoV N protein	Sub-nano-molar concentrations	Not mentioned	Not mentioned	10 min	[96]
Electro-chemical biosensor	Not mentioned	Carbon nanotube	Carbon nanotube FET (CNTFET)	SARS-CoV N protein	5 nM	Not mentioned	Not mentioned	10 min	[97]
	Sputum sample	Not mentioned	ROS based Electro-chemical tracing	Traces of mitochondrial ROS	Less than 500 μL	Not mentioned	97%	Less than 30 s	[98]
	Not mentioned	Not mentioned	Bielectric recognition assay	S1 functional subunit of spike protein of COVID-19	1 fg/mL	Not mentioned	Not mentioned	Not mentioned	[100]
	Not mentioned	ZnO nanowire	Nanowire enhanced EIS biosensing	Spike protein (S1) of SARS-CoV-2	0.4 pg/mL	Not mentioned	Not mentioned	Less than 30 min	[99]
	Clinical sample	Not mentioned	Electrochemical detection	RNA of SARS-CoV-2	200 copies/ml	Not mentioned	Not mentioned	Not mentioned	[100]
	Clinical samples	Not mentioned	Graphene based Field-Effect Transistor (FET)	SARS-CoV-2 spike protein	2.42 × 10^2^ copies/mL	Not mentioned	Not mentioned	Not mentioned	[109]
	Human throat swab specimen	Gold nano-particle	Graphene Field Effect Transistor (FET)	SARS-CoV-2 RNA	2.29 fm	Not mentioned	Not mentioned	Within 2 min	[112]
Smart and wearable biosensor	Exhaled breathing	AuNPs	AI based Smartphone biosensing via hand-based breathalyzer system	SARS-CoV-2	Not mentioned	Not mentioned	Not mentioned	Not mentioned	[124]
Piezoelectric biosensor	Oral swab samples	Nano-particles	Quartz crystal microbalance (QCM)	spike protein of SARS-CoV-2	ng level	Not mentioned	Not mentioned	Not mentioned	[164]
RT-LAMP based biosensor	Oro-pharyngeal swab	Nano-particles	Colorimetric assay	Two target genes i.e., np and F1ab	12 copies per reaction	Not mentioned	Not mentioned	1 h	[21]
Path-Sensor	Not mentioned	Not mentioned	PathSensor based on a genetically modified immune cell	Aerosol detection of SARS-CoV-2	Not mentioned	Not mentioned	Not mentioned	within 3–5 min	[55]

## 3. Role of Nanotechnology in the Advancement of Biosensors

For healthcare and environmental monitoring, biosensors have shown great potential [169]. In the advancement of biosensors, nanotechnology is playing a very important role. With the use of nanomaterials, the working and sensing capacities of biosensors are enriched. Many new signal transduction technologies which have been introduced to biosensors have been facilitated by these nanomaterials. Nanoprobes, nanosensors and nanosystems have allowed fast and simple analyses in-vivo because of their submicron dimensions [170]. A few frequently and conveniently used examples are discussed below.

### 3.1. Gold Nanostructures

Gold-based nanostructures possess extraordinary physicochemical properties which have been mostly implemented in the field of medical care. Gold nanoparticle (AuNP)-based biosensors have been used as signal amplifiers and in resonance light scattering for virus detection [171]. There are several gold nanoparticle-based biosensors for SARS-CoV-2 which provide colorimetric detection of virus. Particularly, thiol-modified antisense oligonucleotides (ASOs) with AuNP capping [AuNP-ASO] is a colorimetric biosensor which has been observed as a successful approach for the detection of SARS-CoV-2 [172] because of its specific approach to the detection of the nucleocapsid phosphor protein (N-gene) in RNA from oropharyngeal swabs and its selective approach with cervical DNA samples toward human papillomavirus. Additionally, this detection works when the targeted RNA sequence of SARS-CoV-2 is present, resulting in the selective gathering of AuNP–ASO nanostructures that give a red-shift in the UV-absorbance spectrum. Moreover, this biosensor can also detect the RdRp gene of SARS-CoV-2 in human nasopharyngeal samples [173]. Additionally, it was found that a lateral flow immunoassay (LFIA) with AuNPs can detect immunoglobulin M (IgM) and IgG antibodies of SARS-CoV-2 in a simultaneous manner with sensitivity 88.66% and specificity 90.63% by using human blood samples [174]. In this study, a nitrocellulose (NC) membrane was used to prepare a test strip on which anti-human-IgG, anti-human-IgM and anti-rabbit-IgG (control) were deactivated along three different test lines. After this, the combination of an AuNP–COVID-19 recombinant antigen conjugate and AuNP–rabbit IgG was used to spray the conjugation pad. It provides the ability to detect the SARS-CoV-2 virus at different stages in patients. Additionally, an enzyme-linked immunoassay (ELISA) with a colloidal gold-immuno-chromatographic (GICA) kit via a rRTPCR-based process is efficient for the detection of SARS-CoV-2 [35]. Moreover, it was 82.4% sensitive in the collaborative GICA-IgM and GICA-IgG (IgM and IgG antibodies) detection and 87.3% sensitive with the ELISA kit. Additionally, for normal patients, both GICA and ELISA were 100% specific [64]. Therefore, it is efficient for COVID-19 detection.

### 3.2. Lanthanide-Doped Polystyrene NPs

Lanthanide-doped NPs show extraordinary optical properties, as they possess unique electronic configurations, including large and sharp emission bands and long luminescence lifetimes, which helps with highly sensitive detection [175]. Further, a lateral flow immunoassay (LFIA)-based biosensor was built by using lanthanide-doped NPs, which can be used for POC diagnosis of viruses [176]. Therefore, a LFIA associated with a lanthanide-doped polystyrene nanoparticles (LNPs)-based biosensor has been developed using the mini-emulsion polymerization technique for SARS-CoV-2 detection [177] which can detect anti-SARS-CoV-2 IgG from a human serum sample within 10 min. Moreover, by using a EDC/NHS chemical reaction of rabbit IgG (R-IgG) and mouse anti-human IgG antibody (MH-IgG), the LNPs were modified to make fluorescent probes. After this, recombinant nucleocapsid phospho-protein, which is responsible for trapping the specific IgG of SARS-CoV-2, was deactivated by using nitrocellulose membrane as a template. It was observed that LFIA and RT-PCR methods provided similar results, except for one sample. It was proven that the LFIA does not show specific results because of the absence of an anti-SARS-CoV-2 IgG standard. (Chen et al., 2020). Therefore, the LFIA method still needs to be explored for the diagnosis of COVID-19 [64,177].

### 3.3. Magnetic Nnanoparticles

Magnetic nanoparticles (MNPs)-based biosensors are useful for the diagnosis of respiratory viruses [178,179]. According to the report of Tian et al., MNPs-based biosensors can be used for the measurement of nucleic acids by using iron oxide NPs (IONPs) and homogeneous circle-to circle amplification (HC2CA) [87]. In this method, individual IONPs and combined IONPs display the specific optical properties (absorption or scattering) under the influence of an external magnetic field. However, the combined IONPs are formed when the detection probes of IONPs are mixed with the HC2CA, giving the end amplicons (ssDNA). Thus, opto-magnetic properties can be analyzed by the states of IONPs. Therefore, this method was found to be specific for distinguishing between SARS-CoV-2 and SARS-CoV gene sequences [87]. Additionally, it was implemented for the detection of RdRp sequences (synthetic complementary DNA) of SARS-CoV-2. Moreover, carboxyl polymer coated magnetic NPs (pcMNPs) were found to be efficient for COVID-19 detection by extracting the RNA, as reported by Zhao et al. [180]. Particularly, this method can provide virus lysis and RNA binding within a single step, and forms a pcMNPs–RNA complex when the RT-PCR technique can identify ORFlab and N genes of the viral RNA [64]. Additionally, magnetic nanosensor-based POC devices were developed by several companies (July 2020), such as T2 Biosystems, Inc. (Lexington, MA, USA) for the COVID-19 pandemic [181]. Hence, this method can detect SARS-CoV-2 [64].

## 4. Conclusions

In conclusion, this review mainly showed that nanobiosensors are one of the great tools for the detection and prevention of SARS-CoV-2, being low cost and high in sensitivity. The outbreak of COVID-19 uncovered the critical need for redesigning clinical diagnostics to actualize new innovations for POC testing with adequate exactness and unwavering quality. Over many years, broad examinations have been undertaken for the delicate identification of infections, and it is obvious from the discussion in this review that nanobiosensing systems have provided tremendous advancements in infection recognition in terms of selectivity, affectability, particularity and reaction time. A number of recent studies have investigated the utility of nanobiosensors for biomedical applications, especially biosensors that are convenient, modest and exceptionally delicate that can be utilized for diagnosing infections or checking their responses to medication. However, currently nanobiosensors can only be effective under exceptionally advanced conditions in a research center. Generally, nanobiosensors pick up analytes in clinical models, sometimes with in-vivo observation. Herein, we have discussed a plasmon-enhanced biosensor, the electrochemical detection of coronavirus with FETs, graphene-based biosensors, optical nanobiosensors, smart and wearables-based biosensors, piezoelectric biosensors, an RT-LAMP biosensor, etc. LSPR was found to be the ideal candidate for the detection of micro and nano-scale analytes. It exhibits great sensitivity to local variations, owing to the enriched plasmonic field in the locality of nanostructures. For the detection of infections induced by coronaviruses such as SARS-CoV, MERS-CoV and SARS-CoV-2, electrochemical methods have revealed their great potential. Graphene is the most attractive material to scientists for the development of new nanobiosensors, due to its cost-effectiveness, high affinity and ease of fabrication. Properties such as sensitivity, robustness, being label-free, being immune to electromagnetic interference and suitability to miniaturization, make optical nanobisensors interesting to scientists. Wearable sensors and smartphone-enabled nanobiosensors were also discussed here. To implement a robust nanobiosensor-based device for the detection of SARS-CoV-2, there is a need for more accurate diagnosing and screening of samples to minimize the false results and reach high standards.

## 5. Future Perspectives

Future work ought to incorporate explicit improvements or blends of different tests, for example, using fast nucleic acid analyses to additionally affirm the test outcome. Current work has endeavored to improve the identification capabilities, straightforwardness and execution of biosensors. There has been much work already done regarding biosensors, and still some work is going on for the development of advanced biosensors, such as LFIA-based biosensors, amplification-free electrochemical CRISPR nanobiosensors and EIS biosensing on µPADs—but still, there are big opportunities with graphene-based biosensors and pathsensors, which are unexplored for COVID-19 testing. Microneedle (MN) technology, which is ideally an arrangement of micro-sized needles on a minute patch, has taken biosensing research to another level. Various MN-based systems have been developed for chronic diseases, but their study and applications to COVID-19 detection are still restricted. These miniature needle arrays can detect biomarkers in/from the skin in a minimally invasive manner to provide (near) real-time diagnosis. Few microneedle devices have been developed specifically for infectious disease diagnoses, though similar technologies are well established in other fields and are generally adaptable for infectious disease diagnosis. These include microneedles for biofluid extraction, microneedle sensors and analyte-capturing microneedles, or combinations thereof. These technologies are in their early stages of development for infectious disease diagnostics, and there is a vast scope for further development [182]. Just this year, wearable device-based detection of COVID-19 was proposed [183]. It can be used for the early detection of asymptomatic and pre-symptomatic cases of COVID-19. Limitations of these studies are: no differentiation of COVID-19 from other viral infections, expensiveness, the need for large datasets, etc. Electronic sensors in, for example, epidermal tattoos, contact lenses, textiles, face masks, wristbands and patches, are currently being explored and in future could be enriched.

## Figures and Tables

**Table 1:** Traditional methods for COVID-19 detection.

Types of Techniques	Name of Detection Techniques	Target	Limit of Detection	Sensitivity	Ref.
ELISA	KT-1033 EDI Novel coronavirus COVID-19 ELISA KIT Platelia SARS-CoV-2 Total Ab assay	IgM/IgG Total antibody against N protein	5 IU/mL -	100% 92.2%	[21]
Luminescent assay	Roche Diagnostics, Elecsys Anti-SARS-CoV-2 Siemens Healthcare, Atellica IM SARS-CoV-2 Total (COV2T) Chemiluminescence, detection kit	Total antibody against N protein Total antibody against RBD of S1 protein IgM and IgG	- - -	100% 100% 100%	[21]
Lateral Flow Immunoassay (LFIA)	National Bio Green Science, NBGC’ Novel Coronavirus(2019-nCoV) IgM/IgG Antibody Rapid Test Kits STANDARD Q COVID-19 Ag test Sure Screen Diagnosis, COVID-19 Rapid Test Cassette	IgM and IgG IgM and IgG IgM and IgG	- - -	100% 88.66% 96%	[21]
Real Time RT-PCR	Xpert Xpress SARS-CoV-2 test Vita PCR SARS-CoV2 assay	N2 and E gene Viral RNA	250 copies/mL 2.73 × 10^0^	100% 100%	[21]
